# Baculovirus Displaying Hemagglutinin Elicits Broad Cross-Protection against Influenza in Mice

**DOI:** 10.1371/journal.pone.0152485

**Published:** 2016-03-29

**Authors:** Sang-Hee Sim, Joo Young Kim, Baik Lin Seong, Huan Huu Nguyen, Jun Chang

**Affiliations:** 1 Graduate School of Pharmaceutical Sciences, Ewha Womans University, Seoul, Korea; 2 Department of Biotechnology, College of Life Science and Biotechnology, Yonsei University, Seoul, Korea; 3 Laboratory of Viral Immunology, International Vaccine Institute, Seoul, Korea; Georgia State University, UNITED STATES

## Abstract

The widespread influenza virus infection further emphasizes the need for novel vaccine strategies that effectively reduce the impact of epidemic as well as pandemic influenza. Conventional influenza vaccines generally induce virus neutralizing antibody responses which are specific for a few antigenically related strains within the same subtype. However, antibodies directed against the conserved stalk domain of HA could neutralize multiple subtypes of influenza virus and thus provide broad-spectrum protection. In this study, we designed and constructed a recombinant baculovirus-based vaccine, rBac-HA virus, that expresses full-length HA of pandemic H1N1 influenza virus (A/California/04/09) on the viral envelope. We demonstrated that repeated intranasal immunizations with rBac-HA virus induced HA stalk-specific antibody responses and protective immunity against homologous as well as heterosubtypic virus challenge. The adoptive transfer experiment shows that the cross-protection is conferred by the immune sera which contain HA stalk-specific antibodies. These results warrant further development of rBac-HA virus as a broad-protective vaccine against influenza. The vaccine induced protection against infection with the same subtype as well as different subtype, promising a potential universal vaccine for broad protection against different subtypes to control influenza outbreaks including pandemic.

## Introduction

Influenza virus is a major respiratory pathogen that causes annual outbreaks and occasional pandemics. Vaccination against influenza is effective way to control the pathogen spread [1]. Production of current licensed influenza vaccines is based on growth of viruses in embryonated chicken eggs that could be problems when there is a high demand for fertilized eggs and risk of egg-related allergy reactions [2]. To prevent accelerating spread of influenza virus, several novel strategies are considered to overcome the egg-dependent production of influenza vaccines. Those include culture-based production of inactivated influenza vaccines [3], recombinant protein-based influenza vaccines [4], influenza DNA vaccines [5], noninfectious influenza virus-like particles [6, 7] and diverse vector systems [8, 9].

Baculovirus has recently been focused as a novel tool for vaccine vector development for several advantages [10]. Baculovirus display system exhibits several advantages as a vaccine vehicle: baculovirus can trigger innate immunity in the hosts, exhibiting strong adjuvanticity itself [11–13]. In addition, the display of antigens on the virion surface makes it readily accessible for immune system to induce protective immunity [14, 15]. Moreover, unlike viral vectors based on mammalian viruses, there is no evidence of preexisting antibodies (Abs) against baculovirus in humans [16]. These attributes have encouraged growing interests to explore baculovirus for vaccine delivery platforms.

Influenza hemagglutinin (HA) is a major target for inducing neutralizing Abs and regarded as one of most crucial components in current influenza vaccines. It is well known that large amounts of the neutralizing Abs are generated against specific antigenic site located in the globular head domain of HA. Recently, a number of studies have shown that HA stalk domain is relatively well conserved and the conserved HA stalk-specific Abs are able to neutralize a broad spectrum of influenza virus subtypes, making it a good candidate for universal vaccine [17–22]. Attempts to develop vaccines that induce high levels of HA stalk-specific Abs have been made. For example, chimeric HA constructs expressing a globular head and a stalk region from different subtypes have recently been developed to produce stalk-reactive Abs that provide broad protection [19, 23, 24].

The objective of this study was to develop a recombinant baculovirus-based vaccine expressing full length of HA (A/California/04/09) protein on its surface envelope (rBac-HA) for a vaccine against broad spectrum of influenza viruses. Here, we showed that two consecutive intranasal (i.n.) immunizations with rBac-HA virus induces significant HA stalk-specific as well as HA head-directed Abs. The rBac-HA vaccination provides protection against challenge with the same subtype as well as cross-protection against other subtype.

## Materials and Methods

### Cell lines

Spodoptera frugiperda 9 (Sf9) cells (Invitrogen, Carlsbad, CA) were propagated at 28°C in SF-900II serum free medium (Gibco BRL, Rockville, MD). Human embryonic kidney 293 cells (ATCC, Manassas, VA) were grown in Dulbecco’s modified Eagle medium (Life Technologies, Gaithersburg, MD) supplemented with 10% fetal bovine serum (FBS). Madin-Darby canine kidney (MDCK) cells (ATCC) were grown in minimal essential medium (MEM) supplemented with 10% FBS.

### Virus strains

The influenza A/California/04/09 virus (pH1N1; mouse adapted) and A/Vietnam/1203/04-PR8/CDC-RG-attenuated (H5N1; mouse adapted) virus were described elsewhere [8]. A/Vietnam/1203/04-PR8/CDC-RG-attenuated is a reassortant virus with the only HA gene of A/Vietnam/1203/04 (H5N1) origin in the genetic background of the high-growth strain A/Puerto Rico/8/34 (H1N1).

### Mice

Female BALB/cAnNCrljOri mice (5 week-old) were obtained from Orient Bio (Seoul, Republic of Korea). Mice were maintained under specific pathogen-free conditions in the experimental facility at the Ewha Womans University. According to the retrospective statistical calculation for this study, more than four mice per each group would be needed to yield a power of 80% (assuming α, 2-tailed, was set at 0.05). This study was carried out in strict accordance with the recommendations in the Institute of Laboratory Animal Resources Guide for the Care and Use of Laboratory Animals. The protocol was approved by guidelines of Ewha Womans University Institutional Animal Care and Use Committee (Permit Number: 2010-9-4). All treatment was performed under anesthesia by isoflurane (Ifran: Hana Pharm, Kyonggi-Do, Korea) inhalation or carbon dioxide euthanasia, and all efforts were made to minimize suffering.

### Construction and purification of HA-displaying baculovirus

The full length HA gene from pH1N1 was synthesized by Bioneer Corp., Daejeon, Korea. HA gene was then amplified by PCR with following primer set: HA forward Sal I 5’GGG TCG ACG CCA CCA TGA AGG CAA TAC TA 3’ and HA reverse Not I 3’ GGG CGG CCG CTT AAA TAC ATA TTC TA 5’ (the restriction sites are underlined). The amplified HA gene was inserted into Sal I and Not I sites of pFastBac dual vector under the control of polyhedrin promoter. The expression cassette was inserted into the baculovirus genome within DH10Bac^™^ (Invitrogen) according to the manufacturer’s protocol (Invitrogen). The recombinant bacmid DNA was rescued and transfected into Sf9 cells and recombinant baculovirus was harvested at 96 h post-transfection. The recombinant baculovirus displaying HA (rBac-HA) was selected by plaque isolation and amplified. The recombinant baculovirus were purified from the final culture supernatants of infected cells by sucrose cushion centrifugation (25% w/w sucrose in 5 mM NaCl, 10 mM EDTA in PBS) using ultracentrifuge (24,000 rpm for 75 min at 4°C). The supernatants were decanted. The pellet was re-suspended in PBS (pH 6.2) and centrifuged for 150 min at 27,000 rpm, 4°C. The pellets were subsequently re-suspended in 0.25 M sucrose and the virus stocks were stored in LN_2_ tank. Purified baculoviruses were titrated by plaque assay using Sf9 cells according to the manufacturer’s protocol (Invitrogen).

### Immunization and virus challenge

Female BALB/c mice were randomized to each group (4~5 mice per group), and intranasally immunized with 1×10^6^, 3×10^6^, 3×10^7^ plaque-forming units (PFU) of rBac-HA virus, intact baculovirus (Bac-control), or PBS. Two weeks after primary immunization the mice were boosted with the same amounts of the vaccines. To assess the protective efficacy of the vaccination, three weeks after the boosting immunization, the mice were infected intranasally with 10 LD_50_ (6 TCID_50_) of pH1N1 or 50 LD_50_ (5.47×10^2^ TCID_50_) of H5N1. Morbidity and mortality were monitored for 14 days after the challenge. According to the humane endpoints, any mice struggling due to infection were euthanized. Briefly, the challenged mice were monitored their body weight twice daily at a 12 hours interval for 14 days. Mice that reduced their body weight under 25% to 0 day post-infection (DPI) were euthanized and spontaneously died mice were removed immediately from the cages: i.e., In the H1N1 and H5N1 challenge study, 1~2 mice died spontaneously at 5 DPI. Surviving mice until 14 DPI were euthanized at the end of the study.

### Western blotting

In order to prepare samples for cross-linking experiment, 3x10^6^ pfu of Bac-control, rBac-HA virus and pH1N1 were treated with a final concentration of 0.5% formaldehyde for 30 min at 4°C under rotation. The samples in loading buffer [50 mM Tris-HCl (pH 6.8), 100 mM dithiothreitol, 2% sodium dodecyl sulfate, 0.1% bromophenol blue, 10% glycerol)] were heated at 65°C (cross-linked samples) or boiled at 100°C (non-cross-linked samples) for 5 min and separated by 8% SDS-PAGE. The gel was transferred to PVDF membrane (Pall, Ann Arbor, MI) in transfer buffer (25 mM Tris, 192 mM glycine, 20% methanol) at 300 mA for 1 h 30 min. The membrane was blocked with TBST buffer containing 5% skimmed milk at 37°C for 1 h and incubated overnight with mouse polyclonal antisera raised against the pH1N1 HA protein at 4°C. After washing, the membrane was incubated with HRP-conjugated rabbit anti-mouse IgG (Abcam, Cambridge, UK) diluted at 1:1000 for 1 h at 37°C. After final washing, proteins were detected using chemiluminescence reagent (GenDEPOT, Barker, TX) and ChemiDOC MP Imaging System with Image Lab Software (Bio-Rad, Hercules, CA).

### Flow cytometry analysis

In order to determine the expression level of HA on the baculovirus envelope, Sf9 cells were infected with rBac-HA virus or Bac-control in 6 well plates at 10 MOI. 48 h post infection, the cells were harvested, washed two times with FACS buffer (0.5% FBS, 0.09% NaN_3_ in PBS) and blocked with anti-mouse CD16/CD32 (Mouse BD Fc block; BD Pharmingen, San Diego, CA) for 5 min at room temperature. After blocking, the cells were incubated with mouse polyclonal anti-HA sera for 40 min at 4°C followed by washing with FACS buffer. The cells were stained with the FITC conjugated anti-mouse IgG (1:100 dilutions) (eBioscience, San Diego, CA). After staining, the cells were washed with FACS buffer. The fluorescence signal was detected using FACS Calibur flow cytometer (BD Biosciences, San Diego, CA) with FlowJo software (TreeStar Inc., Ashalend, OR).

### Enzyme-linked immunosorbent assay

Three weeks after boost immunization, mice were sacrificed and bronchoalveolar lavage (BAL) fluid was collected by flushing lung airway with 1 ml of PBS. The BAL fluid was centrifuged and supernatant were collected for determination of secretory IgA levels. Sera were separated from blood collected from retro-orbital plexus of mice upon anesthesia and stored at -70°C until analysis. HA-specific Ab titers from immunized mice were measured by a direct enzyme-linked immunosorbent assay (ELISA) [25]. Briefly, ELISA plates (F96 Polysorp NUNC immunoplate, Roskilde, Denmark) were coated with purified recombinant HA protein of pH1N1 (50 ng/well) (Immune Technology, New York, NY), or recombinant chimeric H9/1 [H9 HA head on top of an H1 (PR8) stalk] protein (200 ng/well), blocked, and incubated with serially 2-fold diluted sera or BAL samples. As a detection antibody, HRP-conjugated rabbit anti-mouse IgG (Abcam) (1:2000 dilution) or HRP-conjugated goat anti mouse-IgA (Zymed Laboratories, San Francisco, CA) (1:3000 dilution) was used. The plates were developed with 3,3’5,5’-tetramethylbenzidine peroxidase substrate (KPL, Gaithersburg, MD), stopped with 1 M H_3_PO_4_, and analyzed at 450 nm by a Thermo Multiskan^®^ EX (Vantaa, Finland). The end-point titers were determined as the highest serum dilution fold that had reached to a cut-off value by linear regression analysis with R2>0.9, and the cut-off value was set as the sum of the mean blank value plus 3x the standard deviation.

### Chimeric HA protein

The chimeric HA protein containing the stalk domain of H1N1 PR8 and the globular head domain of H9N2 A/guinea fowl/Hong Kong/WF10/99 (cH9/1) was kindly provided by Dr. Peter Palese, Icahn School of Medicine, Mount Sinai, New York, New York [20]. Briefly, High Five cells were infected with recombinant baculovirus expressing cH9/1 at an MOI of 10, harvested 96 h postinfection, and separated from supernatant. For purification of the cH9/1 protein fused with a 6-his tag, the supernatant was collected, incubated with Ni-NTA resin for 2 h at 4°C. The suspension was loaded into column and washed three times. The fractions containing cH9/1 protein was eluted, pooled, and concentrated with a 10 kDa molecular mass cut-off. Final protein concentrations were determined with Bradford reagent.

### Hemagglutination inhibition assay

Three weeks after boost immunization, serum samples were obtained from the retro-orbital plexus, treated with receptor destroying enzyme (RDE, Denka-Seiken, Tokyo, Japan), and inactivated. The samples were serially diluted 2-fold in 96 well V-bottom plate, mixed with an equal volume of influenza virus (8 HA units), and incubated. Followed by adding of 0.5% chicken red blood cells in Alsever’s solution (Sigma Aldrich, St. Louis, MO), the plates were gently mixed and incubated. The HI titer was determined by the reciprocal of the last serial dilution point that contained non-agglutination reaction. Serum hemagglutination inhibition (HAI) Ab titers of ≥1:40 correlate with protection at least 50% against infection [26].

### Lung virus titer

Five days after challenge with influenza A virus, mice from each group were sacrificed and the lungs tissues were harvested. The tissues were homogenized through a 70 μm cell strainer (BD Labware, Franklin Lakes, NJ) with 3 ml serum-free MEM. Following centrifugation, the supernatants were collected and viral titers were determined by plaque assay on MDCK cells. The data were expressed as PFU per gram of lung tissue and the lower limit of detection of the assay was 10^2^ PFU/gram.

### Adoptive transfer study

BALB/c mice were immunized twice with 3×10^7^ PFU of rBac-HA virus or control-Bac via intranasal route. Three weeks later mice were sacrificed, and sera, BAL fluids and splenocytes were collected. The pooled immune sera and BAL fluids were heat-inactivated for 30 min at 56°C, and BAL fluids were concentrated to 10-fold. Single cell suspensions from pooled spleen were prepared in 200 μl containing 5×10^7^ cells. Antibody titers against whole HA or HA stalk region were measured from the sera and BAL fluids of the donor mice by ELISA. In order to measure the magnitude and functionality of the HA-specific CD8^+^ T cells from the donor, tetramer or intracellular IFN-γ positive cells were measured by flow cytometry. Naïve recipient BALB/c mice received 200 ml of pooled immune sera or cells via intravenous route. Fifty microliter of concentrated BAL fluids was intranasally injected into recipient mice. Six hours later the recipient mice were challenged with 10 LD_50_ (1.09×10^2^ TCID_50_) of H5N1 influenza virus. Morbidity and mortality were monitored for 14 days after the challenge.

### Tetramer and intracellular cytokine staining

For tetramer staining, cell suspensions obtained from peripheral blood or lung tissue were blocked with anti-mouse CD16/32 and stained with anti-CD44 FITC (clone IM7; BioLegend, San Diego, CA), anti-CD8 PE-Cy5 (clone 53–6.7; BioLegend), anti-CD45 APC (clone 30-F11; BioLegend) and MHC class I tetramer HA_533-541_ (H-2K^d^/IYSTVASSL).

For intracellular staining, the cell suspensions were incubated for 6 hours with HA_533-541_ peptide/recombinant human IL-2 (BioLegend) or phorbol myristate acetate (PMA)/ionomycin in Iscove’s Modified Dulbecco’s Media containing 10% FBS and Brefeldin A (eBioscience). Following incubation, the cells were blocked with anti-mouse CD16/32, surface stained with anti-CD44 FITC, anti-CD8 PE-Cy7 and anti-CD45 APC, and fixed in BD FACS lysing solution (BD Pharmingen). The fixed samples were permeabilized in FACS buffer containing 0.5% saponin and stained with anti-IFN-γ PE (clone XMG1.2; eBioscience).

### Statistical methods

All data were plotted as mean±standard error (n = 4~5). Statistical Analyses were performed using GraphPad Prism version 6.07 (GraphPad Software, Inc., La Jolla, CA). Groups in each experiment were compared by an unpaired, two-tailed Student’s *t*-test, one-way ANOVA, and repeated measures ANOVA, respectively. The results were followed up by post hoc analysis using Bonferroni’s procedure and the difference was considered statistically significant when P value < 0.05.

## Results

### Generation of recombinant baculovirus expressing pH1N1 HA

**Fig 1:**
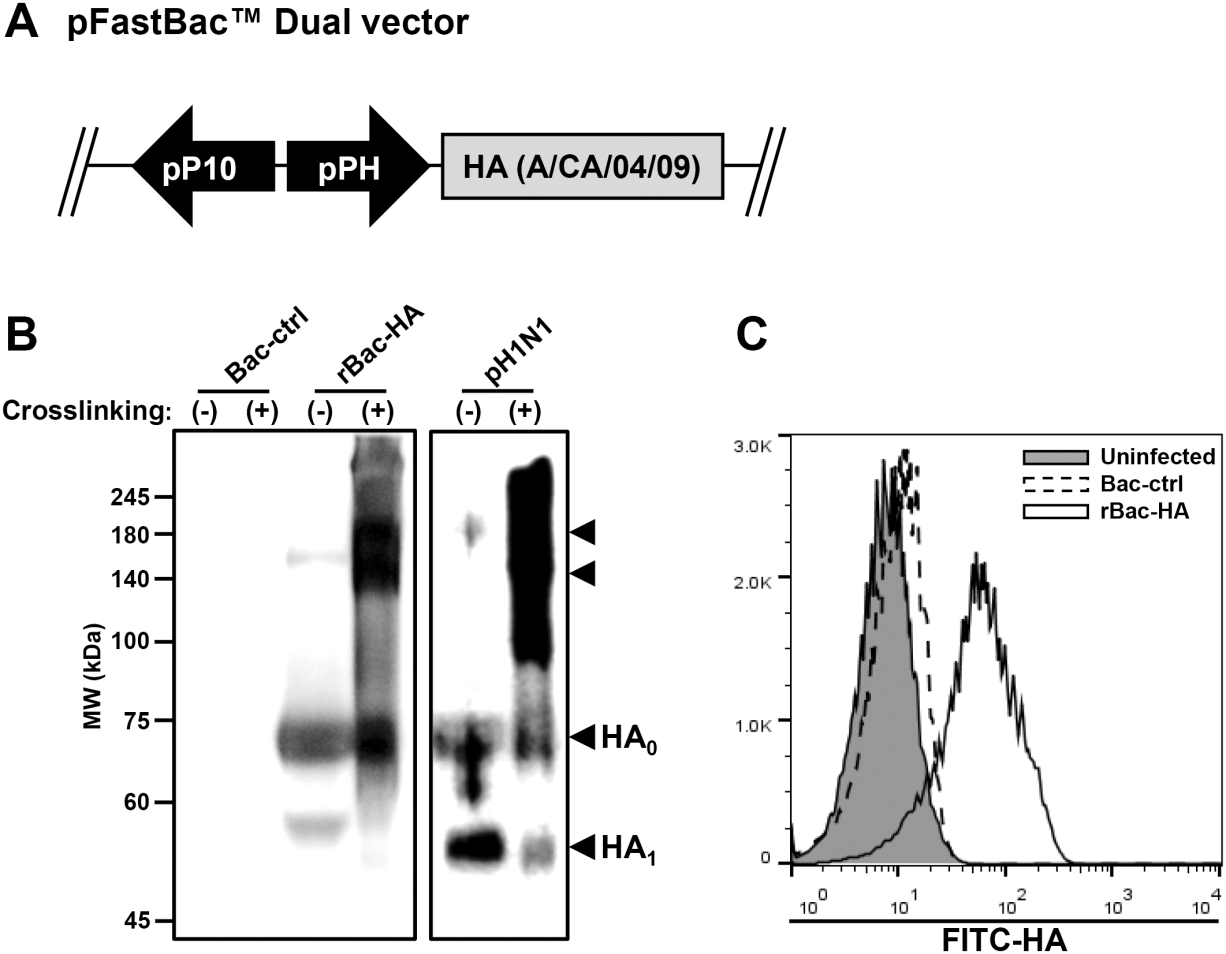
Construction and characterization of rBac-HA virus. (A) A schematic diagram of baculovirus vector construct containing HA gene. The pFastBac dual vector has engineered to encode pH1N1 HA gene from A/California/04/09 under the control of the polyhedrin (PH) promoter. The recombinant baculovirus, rBac-HA virus, was generated using the Bac-to-Bac baculovirus expression system. (B) The presence of multimeric HA protein in the purified viral particle was confirmed by cross-linking and western blotting as described in the Materials and Methods (HA proteins indicated by the arrowhead). Virus particles corresponding to 3×10^6^ PFU were cross-linked, heated in loading buffer at 65°C (cross-linked samples) or 100°C (non-cross-linked samples), and loaded for each lane. (C) The expression level of HA protein on the baculovirus envelope was analyzed using flow cytometry. The Sf9 cells were infected with rBac-HA virus or Bac-control at MOI of 10. 48 h post-infection, the cells were analyzed by HA-specific polyclonal antibody to determine the expression of rBac-HA on the envelope. Uninfected cells used as a negative control. *Bac-ctrl*, Bac-control-infected cells; *rBac-HA*, rBac-HA-infected cells; *Uninfected*, uninfected cells.

The recombinant baculovirus expressing pH1N1 HA, rBac-HA, was generated using Bac-to-Bac system with a pFastBac dual vector in which the full length sequence of A/California/04/09 HA was inserted under the control of polyhedrin promoter (Fig 1A). To confirm the presence of pH1N1 HA in the rBac-HA virion particles, the sucrose gradient-purified baculovirus particles were examined by western blotting analysis using the polyclonal antibody raised against the HA. Specific band of HA0 (HA precursors) at molecular weight of approximately 70 kDa was detected in the sucrose gradient-purified rBac-HA particles, since insect cells express low levels of furin-like enzymes and the HA0 was not fully cleaved into HA1 and HA2 subunits [15, 27]. In the purified influenza virus (A/California/04/09) particle, HA0 as well as cleaved HA1 subunit (approximately 50 kDa) was observed. To investigate whether HA proteins in the rBac-HA particles are present in the oligomeric structure, cross-linking with formaldehyde was performed and the cross-linked HA was observed as a mixture of monomer (70 kDa), dimer (140 kDa) and trimer of HA0 (210 kDa), respectively. Cross-linked A/California/04/09 particles exhibited more diverse oligomeric HA structures than the cross-linked rBac-HA. The purified Bac-control particle as a negative control showed no specific band (Fig 1B). To further confirm the display of HA protein on the cell surface, Sf9 cells were infected with rBac-HA virus and 48 h later the cells were analyzed by flow cytometry following surface staining with HA-specific Abs. As shown in Fig 1C, fluorescence intensity was specifically increased on rBac-HA virus-infected Sf9 cells compared to the Bac-control-infected or uninfected cells. Together, these results indicate that A/California/04/09 HA is successfully displayed on the cell surface as well as on the rBac-HA baculovirus particles.

### Intranasal immunization with rBac-HA virus elicits robust humoral immune response

**Fig 2:**
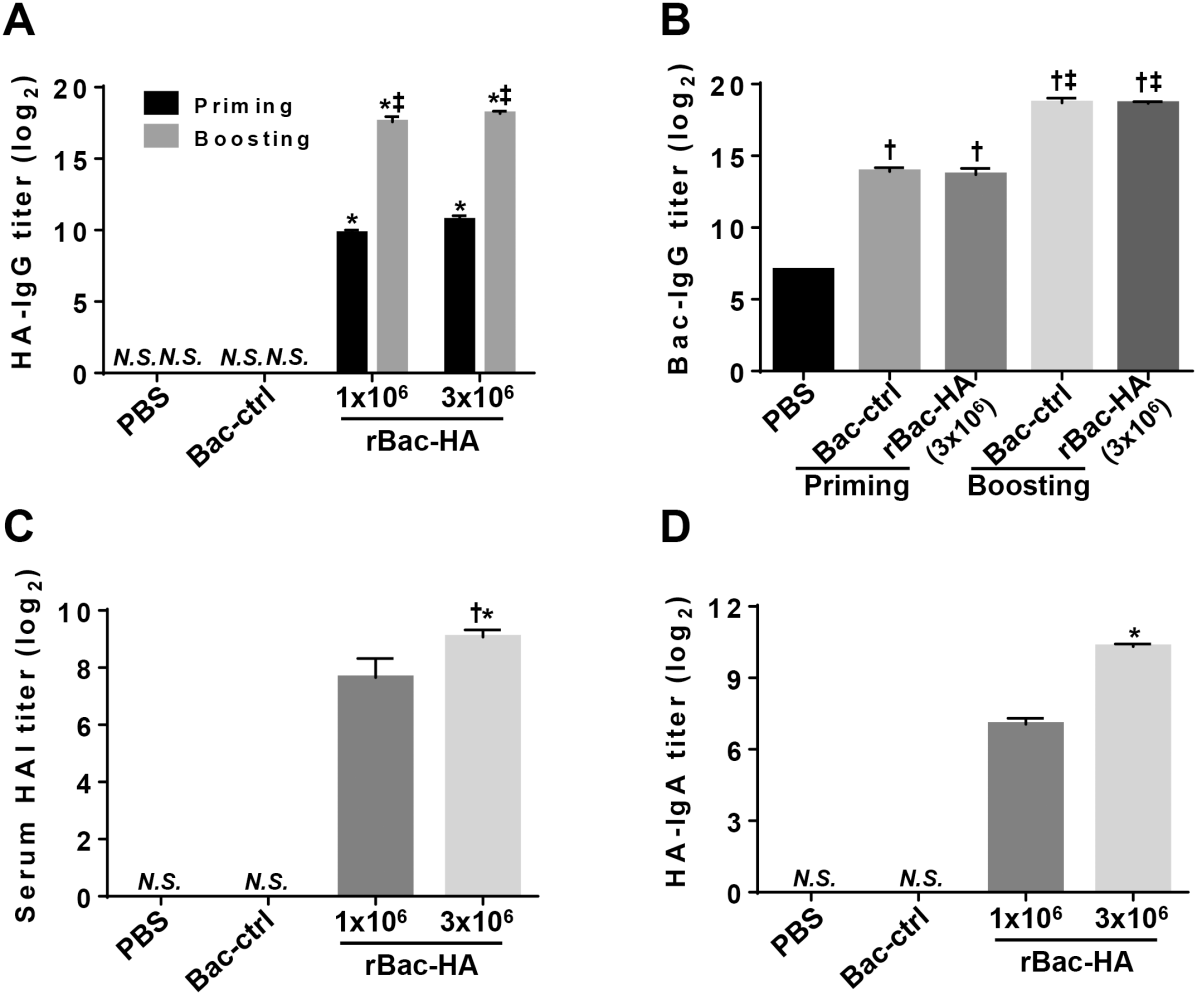
Humoral immune response induced by intranasal rBac-HA virus immunization. BALB/c mice (n = 4/group) were immunized twice on day 0 and 16 with 1×10^6^ PFU or 3×10^6^ PFU of rBac-HA virus via intranasal route. Sera were collected from primed and boosted mice 2 and 3 weeks after vaccination, respectively. Control mice were immunized i.n. with 3×10^6^ PFU of Bac-control. The group of mice injected with PBS as negative control. (A) HA-specific and (B) Baculovirus-specific IgG antibody titers were measured in primed and boosted mice sera by ELISA, respectively. (C) Hemagglutination inhibition (HAI) titers against A/California/04/09 were measured in the sera obtained from boosted mice. (D) Mucosal HA-specific IgA in BAL fluid were measured 3 weeks after vaccination by ELISA. The results indicate Log_2_ end-point titers. “N.S.” indicates that there is no statistical significance between “PBS” group and “Bac-ctrl" group. *, Statistical significance with “Bac-ctrl” group (p<0.05). ^‡^, Statistical significance with “Priming” group (p<0.05). ^†^, Statistical significance with “PBS” group (p<0.05). *N*.*S*., not significant; *Bac-ctrl*, Bac-control.

To evaluate the immunogenicity of rBac-HA, the groups of mice were inoculated via i.n. route with either 1×10^6^ or 3×10^6^ PFU of rBac-HA virus. As a control, mice immunized with 3×10^6^ PFU of Bac-control were included. Two weeks after primary immunization, the mice were boosted with the same doses and the sera were collected at week 2 and 5. As shown in Fig 2A, moderate levels of the HA-specific IgG response were detected in the sera obtained from rBac-HA virus-immunized mice at 2 weeks after priming. However, the levels of such Abs were significantly increased 3 weeks after the boost immunization. There was no significant difference in the Ab levels between two different doses of rBac-HA virus. PBS and Bac-control mice showed no detectable HA-specific IgG titer (Fig 2A). All group of mice injected with baculovirus induced carrier-specific antibody responses (Fig 2B).

To determine whether the serum Abs have ability of inhibiting hemagglutination, we performed HAI assay. Two weeks after priming, HAI levels to A/California/04/09 viruses in the sera were undetectable or low (<1:20) (data not shown). However, 3 weeks after boost immunization, the sera of mice vaccinated with 3×10^6^ PFU and 1×10^6^ PFU of rBac-HA virus showed an average HAI titer of 1:640 and 1:160, respectively. As expected, either Bac-control or PBS group showed no significant HAI titer (Fig 2C).

Next, HA-specific IgA in BAL collected at 3 weeks after boost immunization was determined by ELISA. The results showed that HA-specific IgA was detected in BAL fluid of mice immunized with 1×10^6^ PFU and 3×10^6^ PFU of rBac-HA virus, respectively (Fig 2D). No detectable HA-specific IgA was observed in the BAL fluid of the control groups. Taken together, these results showed that intranasal immunization with rBac-HA virus induces significant HA-specific humoral responses in the systemic as well as mucosal compartments.

### rBac-HA virus vaccination confers protection against homologous pH1N1 challenge

**Fig 3:**
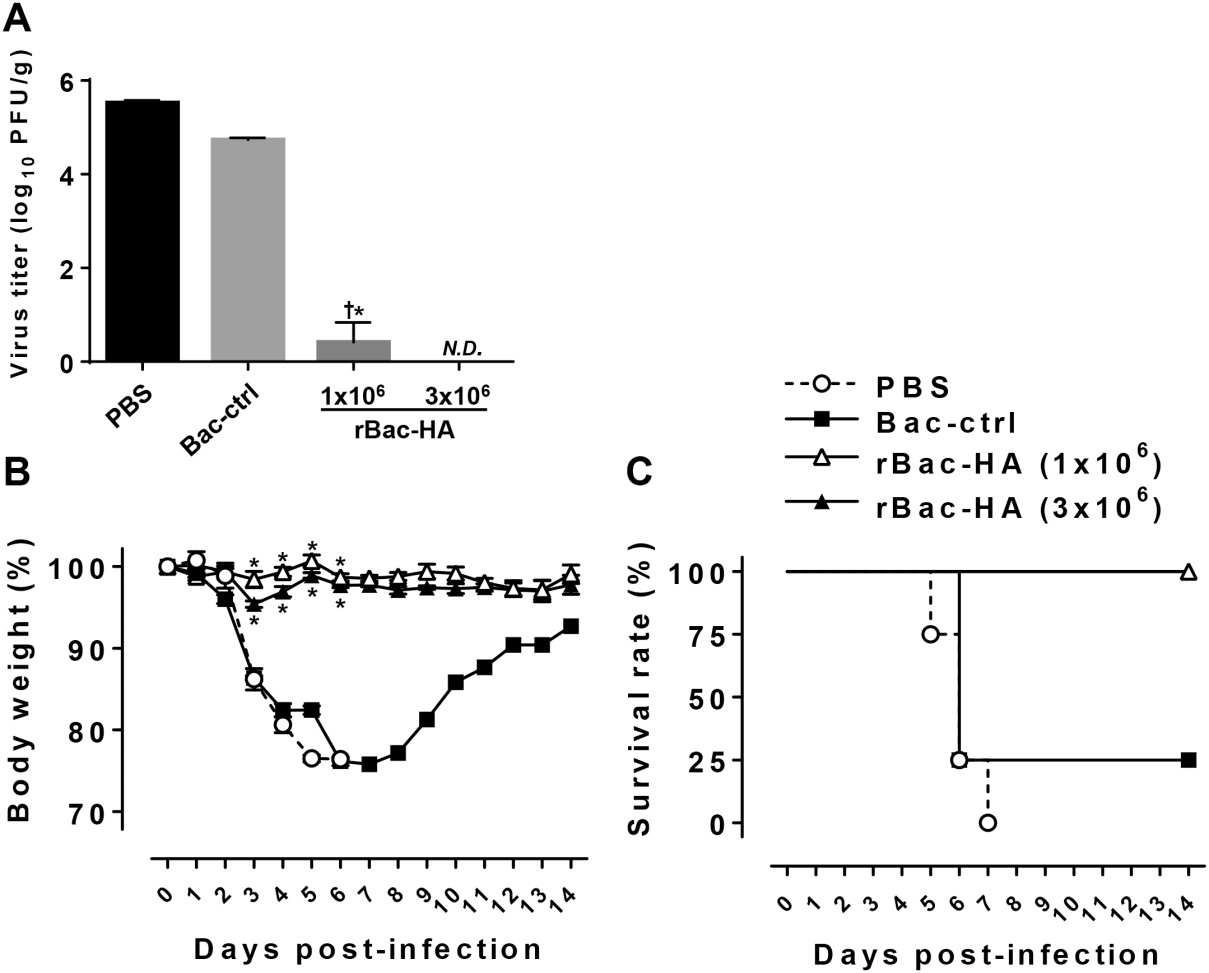
Protection from A/California/04/09 homologous influenza virus challenge by intranasal vaccination with rBac-HA virus. BALB/c mice (n = 4/groups) were injected intranasally with 1×10^6^ or 3×10^6^ PFU of rBac-HA virus on day 0 and 16. Control mice were immunized i.n. with 3×10^6^ PFU of Bac-control. Each group of mice was challenged intranasally with 10 LD_50_ of A/ California /04/09 virus. (A) Viral titers in the lung at day 5 after challenge was measured by standard plaque assay with Madin-Darby canine kidney cells. The results were expressed in terms of log_10_ of 4 mice per group. (B) Challenged mice were monitored for weight loss during 14 days. (C) Survival of all group of mice were also monitored for 14 days. The results were expressed in percent body weight compared to beginning of the trial. ^†^, Statistical significance with “PBS” group (p<0.05). *, Statistical significance with “Bac-ctrl” group (p<0.05). *N*.*D*., not detected; *Bac-ctrl*, Bac-control.

To test whether the rBac-HA virus immunization protects mice against infection with homologous influenza virus, the vaccinated and control mice were challenged with 10 LD_50_ of A/California/04/09 virus three weeks after the last immunization. All mice were sacrificed at day 5 post challenge to determine the virus titers in the lungs. Mice vaccinated with rBac-HA virus had no detectable virus (<10 PFU/g) in the lungs. In contrast, the control mice exhibited high viral load in their lungs (~1×10^6^ PFU/g) (Fig 3A). Whereas mice receiving Bac-control or PBS experienced severe weight loss and exhibited only 25% and 0% survival, respectively, all mice immunized with rBac-HA virus were protected from death without significant weight loss after challenge (Fig 3B and 3C). These results indicate that i.n. immunization with rBac-HA vaccine confers complete protection against lethal infection with homologous influenza virus.

### rBac-HA vaccination induces HA stalk-specific antisera and cross-protection against infection with heterosubtypic influenza virus

**Fig 4:**
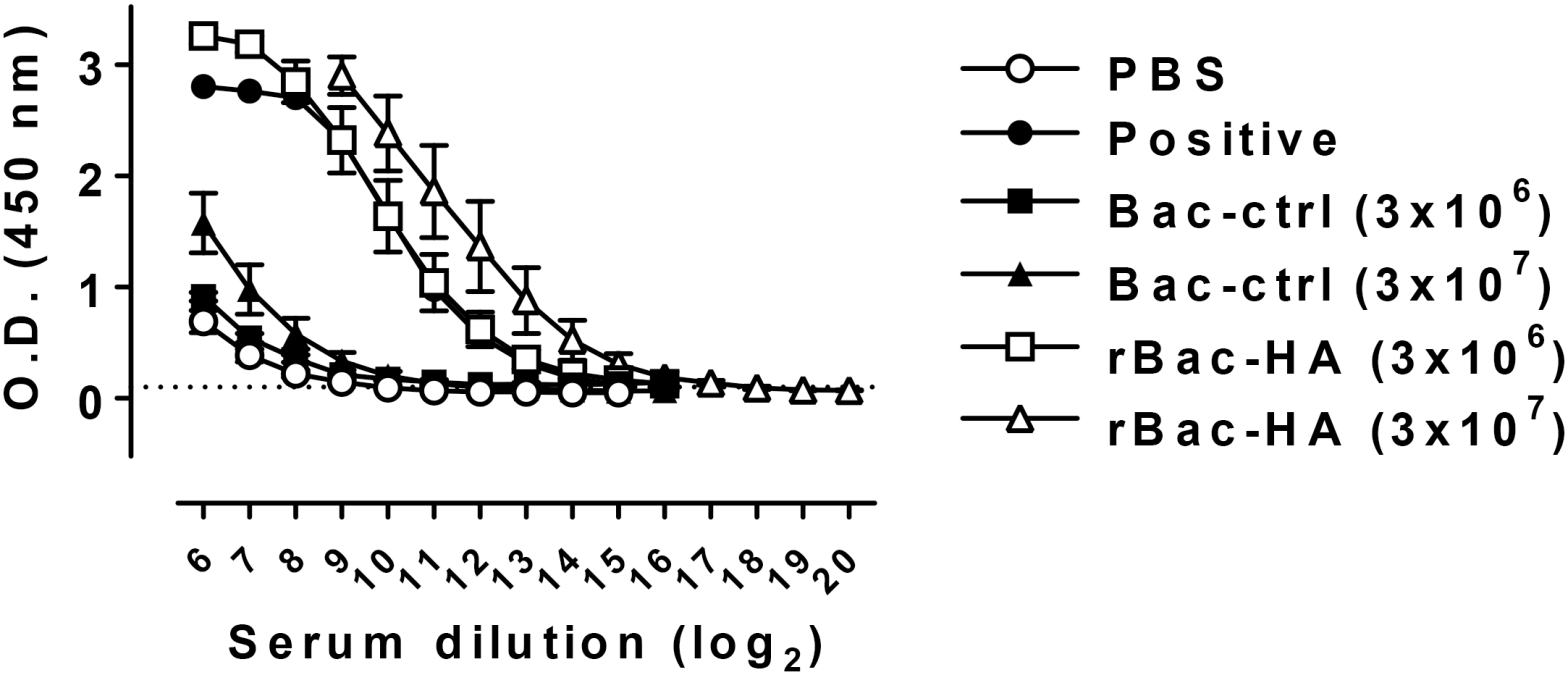
rBac-HA virus elicited stalk-specific Abs. BALB/c mice (n = 5/group) were immunized twice on day 0 and 16 with 3×10^6^ PFU or 3×10^7^ PFU of rBac-HA virus via intranasal route. Sera were collected from all mice 3 weeks after second vaccination. HA stalk-specific Abs were measured in boosted mice by ELISA using chimeric H9/1 protein [H9 HA head on top of an H1 (PR8) stalk domain]. The result was expressed absorbance at 450 nm. *O*.*D*., optical density; *Positive*, Sera from mice vaccinated with 3×10^7^ PFU of recombinant adenovirus encoding HA of H5N1 virus and ectodomain of matrix 2 protein (M2e) of H1N1 virus; *Bac-ctrl*, Bac-control.

To investigate whether stalk-specific Abs could be induced by our rBac-HA vaccine construct, sera from the vaccinated mice were collected and tested for reactivity to recombinant cH9/1 protein by ELISA. Pica and her colleagues suggested that the cH9/1 was prepared by taking advantage of a disulfide that exists between cysteines 52 and 277 in the HA protein and by exchanging the intervening sequence with that from a different HA subtype. By selecting HA heads against which most of the human population is naïve and uncommon among laboratory strains, serum antibodies that are reactive with the chimeric constructs are likely directed to the stalk region. They also showed that the cH9/1 protein reacted specifically with antibodies for the H9 head and H1 stalk except antibodies against the globular head domains of H6 and PR8, and stalk domain of H3 [18]. The results show that mice immunized with rBac-HA virus (3×10^6^ PFU and 3×10^7^ PFU) elicited significant levels of specific Abs to chimeric H9/1 antigen after boost immunization (Fig 4). As negative controls, Bac-control groups did not show any reactivity to HA stalk region. Sera from mice vaccinated with 3×10^7^ PFU of recombinant adenovirus encoding HA of H5N1 virus and ectodomain of matrix 2 protein (M2e) of H1N1 virus, which induced significant level of HA stalk-reactive Abs [18, 20], were used as a positive control (Fig 4). Mice vaccinated with higher dose of rBac-HA virus (3×10^7^ PFU) exhibited 4-fold higher level of chimeric H9/1-specific Abs compared to that immunized with lower dose of rBac-HA virus (3×10^6^ PFU) suggesting that generation of stalk-specific Abs is dose dependent. Taken together, we found that rBac-HA vaccination induced simultaneously HA head-directed as well as HA stalk-specific antibody responses. The latter might be involved in cross-protection against infection with heterosubtypic influenza virus.

**Fig 5:**
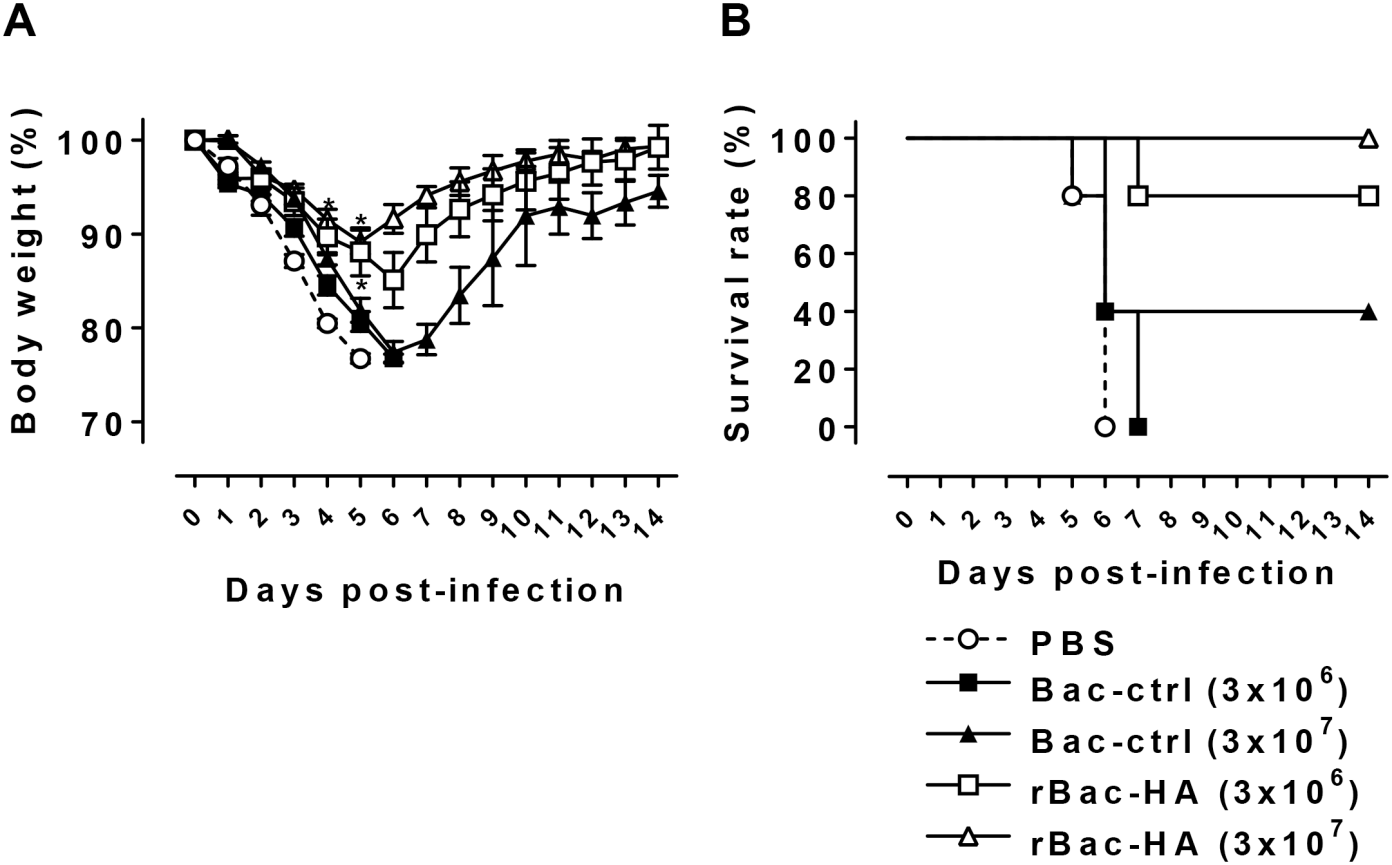
Mice immunized with rBac-HA virus show protection against challenge with heterosubtypic H5N1 virus. BALB/c mice (n = 5/group) were immunized twice on day 0 and 16 with 3×10^6^ PFU or 3×10^7^ PFU of rBac-HA virus via intranasal route. Each group of mice was challenged by intranasal administration with 50 LD_50_ of H5N1 influenza virus. (A) Weight loss was monitored daily for 14 days after challenge. The results were expressed in percent body weight compared to beginning of the trial. (B) Survival of all group of mice were also monitored for 14 days. *, Statistical significance with “Bac-ctrl” group (p<0.05). *Bac-ctrl*, Bac-control.

Since i.n. immunization with rBac-HA virus induced significant HA stalk-specific Ab responses in the previous experiments, we next asked whether i.n. immunization with our rBac-HA construct induces cross-protection against heterosubtypic influenza challenge. To this end, the immune mice were challenged with high dose (50 LD_50_) of H5N1 virus that shares the same HA stalk of group 1 viruses. Mice immunized with 3×10^6^ PFU of rBac-HA virus experienced ~15% weight loss by day 6 post-challenge but rapidly recovered, and exhibited 80% survival rate (Fig 5). The group of mice immunized with 3×10^7^ PFU of rBac-HA virus experienced ~10% weight loss and showed complete protection from the challenge. The control mice receiving Bac-control or PBS experienced severe weight loss and did not survive the challenge (Fig 5). Taken together, our results indicate that i.n. immunization with rBac-HA vaccine provides protection against homologous as well as heterosubtypic influenza virus infections and the vaccine-induced HA stalk-specific Abs may contribute to the cross-protection.

### Adoptive transfer of rBac-HA virus-vaccinated mice sera confers cross-protection against heterosubtypic influenza challenge

**Fig 6:**
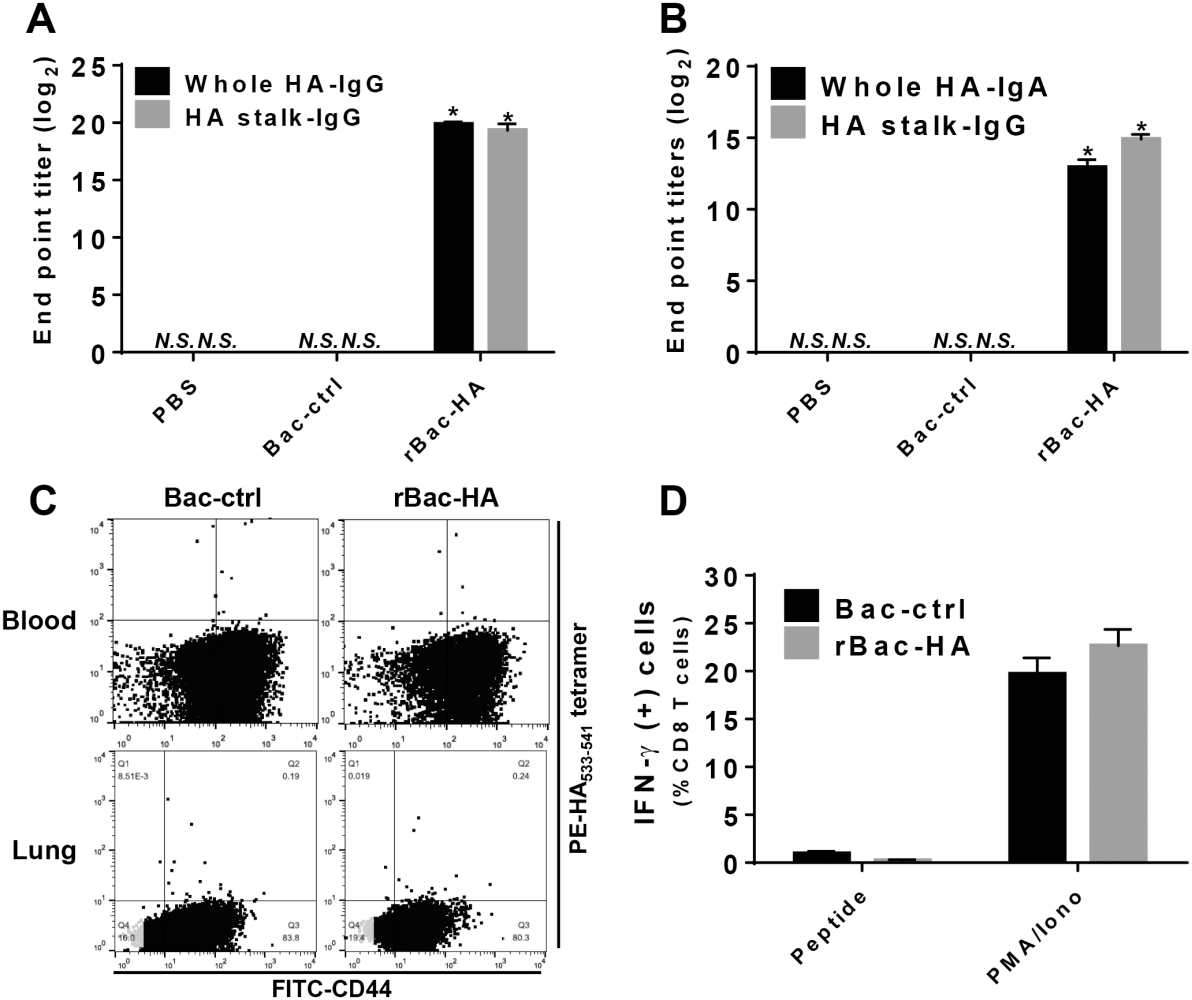
Sera, BAL fluids and splenocytes from rBac-HA virus-immune mice for adoptive transfer study. BALB/c mice (n = 5/group) were immunized twice on day 0 and 16 with 3×10^7^ PFU of rBac-HA virus via intranasal route. Sera, BAL fluids and splenocytes were collected from the immunized mice 3 weeks after second vaccination. (A) Whole HA or HA stalk protein-specific IgG in the pooled sera and (B) HA-IgA or HA stalk-IgG in the pooled BAL fluids were measured by ELISA. The results indicate Log_2_ end-point titers. (C) HA-specific CD8^+^ T cells (H-2K^d^/HA_533-541_ tetramer^+^, CD8^+^ and CD44^+^) were obtained from peripheral bood or lung tissue of boosted mice, and measured by flow cytometry. (D) Intracellular IFN-γ-producing CD8^+^ T cells (IFN-γ^+^, CD8^+^ and CD44^+^) from the donor lung tissue were measured by flow cytometry. “N.S.” indicates that there is no statistical significance between “PBS” group and “Bac-ctrl" group. *, Statistical significance to “Bac-control” (p<0.05). *N*.*S*., not significant; *Bac-ctrl*, Bac-control.

**Fig 7:**
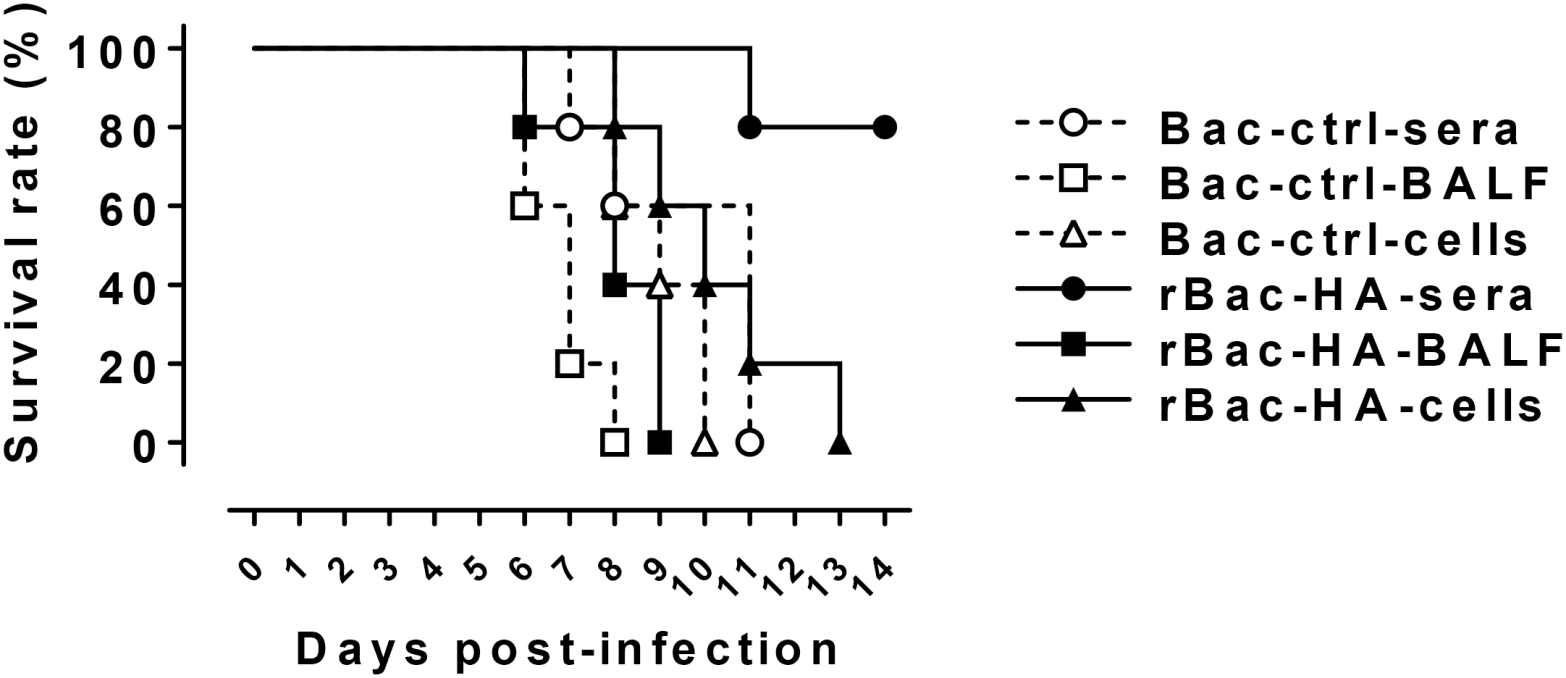
Adoptive transfer of sera from rBac-HA-immune mice provides protection against challenge with heterosubtypic H5N1 virus. BALB/c mice (n = 5/group) were immunized twice on day 0 and 16 with 3×10^7^ PFU of rBac-HA virus via intranasal route. Sera, BAL fluids and splenocytes collected from the immunized mice 3 weeks after second vaccination were adoptively transferred to naïve mice, and 24 h later these mice were challenged with 10 LD_50_ of H5N1 influenza virus. Survival of recipient mice were monitored for 14 days after challenge. *Bac-ctrl*, Bac-control.

In order to determine which factors are associated with heterosubtypic immunity in the rBac-HA virus-vaccinated mice, adoptive transfer study was performed. In the pooled sera from mice immunized twice with 3×10^7^ PFU of rBac-HA virus, significantly increased HA head-directed as well as HA stalk-specific antibody responses were observed (Fig 6A). Both HA-specific IgA and HA stalk-IgG were also increased in the BAL fluids from rBac-HA virus-immunized mice (Fig 6B). However, HA-specific CD8^+^ T cells were not detected in the blood or lung tissues of the rBac-HA virus and Bac-control immunized mice by HA_533-541_ tetramer or intracellular IFN- γ staining (Fig 6C and 6D), even though it is possible that other HA-specific CD8^+^ T cells might be mounted toward unknown minor epitopes. After influenza virus challenge, naive mice that received passively transferred immune sera from rBac-HA virus-immunized mice exhibited 80% survival after lethal infection (10 LD_50_) of heterosubtypic H5N1 virus (Fig 7). In contrast, passive transfer of BAL fluids or splenocytes from the rBac-HA virus-immunized mice had no effect on protection against the H5N1 challenge (Fig 7). These results clearly demonstrated that rBac-HA-immune sera, not BAL fluids or splenocytes, mediates the cross-protection against heterosubtypic influenza virus infection.

## Discussion

We describe a novel recombinant baculovirus-based influenza vaccine candidate which displays full-length HA protein derived from A/California/04/09 virus and show that i.n. immunization with this vaccine induces HA-specific humoral responses along with protection against homosubtypic and heterosubtypic challenge. Importantly, high levels of Abs directed against the stalk region of the HA were induced upon immunization with rBac-HA virus. In the previous studies, chimeric HAs that consist of globular head and stalk domains from different influenza virus strains have been generated, and various heterologous vaccination protocols have been employed to elicit high titers of stalk-specific Abs selectively [19, 23, 28]. To our surprise, our vaccine candidate rBac-HA virus generated significant level of HA stalk-specific Abs by two consecutive homologous immunizations. To our knowledge, this is the first report showing that HA stalk-specific Abs could be induced by homologous vaccination, instead of heterologous boosting with different chimeric HA molecules [19, 23]. This might be due to the mucosal route of vaccination, since it has been reported that intranasal injection could bypass pre-existing vector immunity and enhance immune responses in mice [29, 30]. Consistently, mice vaccinated with rBac-HA virus could be cross-protected from lethal heterosubtypic challenge with H5N1influenza virus.

Previously, several groups have demonstrated that recombinant baculovirus system could be used to display full-length proteins and domains of proteins as fusions on the surface of virion [31, 32], and viral particles containing these foreign proteins have been successfully used as immunogens [14, 15, 33]. Especially, live recombinant baculovirus expressing HA could be implicated as an effective vaccine candidate against influenza virus infection [34, 35]. As foreign antigen being displayed on the viral surface, it might be easily accessible to immune recognition components. We expect that the HA protein precursor displayed on the rBac-HA particle retains the prefusion configuration of HA2 and is more likely to elicit antibodies which bind to the stalk and provide broad neutralization. In this regard, it is interesting to note that influenza virus subviral particles stripped of HA1 from influenza virions are capable of eliciting HA2-specific antisera, but only to the extended, low pH conformation [36]. This led us to question how stalk-specific Abs could be successfully induced by consecutive immunization with our baculovirus-based vaccine containing the homologous HA protein. We propose the following hypotheses that presently explain our findings. First, innate immunity elicited by baculovirus itself may enhance broader antigen-specific B-cell proliferation and differentiation including stalk-specific memory B cells. It is known that baculovirus stimulates Toll-like receptor (TLR) family such as TLR3 and TLR9 [37, 38]. Several reports have extended the evidences that TLR might provide efficient B-cell activation and enhance antibody responses dependent on the type of antigen [39–42]. Secondly, it is likely that HA displayed on the baculovirus surface is presented in its oligomeric form resulting in optimal immunogenicity, since oligomerization is required for efficient transport of the HA proteins to the host cell membrane [43]. Our cross-linking experiment with purified rBac-HA particle supports this hypothesis (Fig 1). In addition, different glycosylation in the insect cells may have altered the relative immunogenicity of HA1 region to HA2, leading to higher responses to HA2 stalk region. This hypothesis has been supported by the studies showing that the differential state of HA molecules expressed in insect cells such as glycosylation and/or oligomerization influences the recognition of innate immune receptors [44] as well as antibody responses to epitopes in the different regions [45]. In this regard, our results also emphasize the importance of future studies for structure-based characterization and improvement in HA-stalk targeted universal vaccine. Thirdly, it is possible that the characteristics of pH1N1 HA is associated with preferred priming of HA-stalk-specific antibody responses. It has been recently reported that infection with the highly novel pH1N1 influenza strain in humans have substantially increased activated plasmablasts targeting to the HA stalk region [46–48]. It has been also proposed that the majority of HA trimers on 2009 pH1N1 virus may have sufficient space to bind anti-stalk Abs that target conserved HA epitopes [49]. It will be of interest to study these various possibilities in the future.

Previously, it has been shown that baculovirus itself has the ability to stimulate strong innate immunity. Inoculation of wild-type baculovirus alone can stimulate the secretion of inflammatory cytokines from innate immune cells and confer protection from lethal virus infection in mice [37]. Consistent with this report, intranasal inoculation of control baculovirus also provided partial protection against influenza virus challenge in our study (Figs 3 and 5). The baculovirus-associated protection might be involved with the activation of innate immunity through the induction of type I interferons by both TLR9-dependent [50] and TLR9-independent signaling [51].

In conclusion, intranasal vaccination with rBac-HA virus induced complete protection against homosubtypic challenge. Also, we showed that rBac-HA vaccination provides substantial cross-protection against heterosubtypic challenge, which might be mediated by HA stalk-specific antibody responses. The most notable finding in this study is that HA stalk-specific Abs can be induced by homologous vaccination with rBac-HA virus. Since HA stalk-specific Abs are known to be protective against a variety of influenza viruses, further investigation into the mechanism involved in this observation would be helpful in the development of a universal influenza vaccine. Our baculovirus-based vaccine candidate offers a new strategy for development of a universal influenza vaccine.

## Supporting Information

S1 ARRIVE Checklist (PDF)
